# Development of prefrontal cortex

**DOI:** 10.1038/s41386-021-01137-9

**Published:** 2021-10-13

**Authors:** Sharon M. Kolk, Pasko Rakic

**Affiliations:** 1 Department of Molecular Neurobiology, Donders Institute for Brain, Cognition and Behaviour and Faculty of Science, Radboud University, Nijmegen, The Netherlands; 2 Department of Neuroscience and Kavli Institute for Neuroscience, Yale University, New Haven, Connecticut USA

**Keywords:** Developmental biology, Neuroscience

## Abstract

During evolution, the cerebral cortex advances by increasing in surface and the introduction of new cytoarchitectonic areas among which the prefrontal cortex (PFC) is considered to be the substrate of highest cognitive functions. Although neurons of the PFC are generated before birth, the differentiation of its neurons and development of synaptic connections in humans extend to the 3rd decade of life. During this period, synapses as well as neurotransmitter systems including their receptors and transporters, are initially overproduced followed by selective elimination. Advanced methods applied to human and animal models, enable investigation of the cellular mechanisms and role of specific genes, non-coding regulatory elements and signaling molecules in control of prefrontal neuronal production and phenotypic fate, as well as neuronal migration to establish layering of the PFC. Likewise, various genetic approaches in combination with functional assays and immunohistochemical and imaging methods reveal roles of neurotransmitter systems during maturation of the PFC. Disruption, or even a slight slowing of the rate of neuronal production, migration and synaptogenesis by genetic or environmental factors, can induce gross as well as subtle changes that eventually can lead to cognitive impairment. An understanding of the development and evolution of the PFC provide insight into the pathogenesis and treatment of congenital neuropsychiatric diseases as well as idiopathic developmental disorders that cause intellectual disabilities.

## PFC definition

There is little disagreement that the human cerebral cortex is the organ that enabled abstract thinking and the creation of civilization, including architecture, science and all types of art. Using a wide variety of methodologies, the size and cytoarchitecture of the frontal lobe, and more specifically the PFC, has been extensively studied over the years in various species. The PFC in humans and nonhuman primates can be divided into a collection of structurally and functionally different subdomains positioned anterior to the motor cortex; the medial (mPFC), lateral prefrontal cortex (lPFC) and orbitofrontal cortex (oFC). The lPFC is mostly involved in language and executive processing, while the oFC and mPFC are known to contribute to cognitive functioning and emotional control [1–4]. The mPFC can be further subdivided into the infralimbic (IL), the prelimbic (PL) and anterior cingulate cortex (ACC). The most ventral subdomain of the mPFC is the infralimbic cortex (IL) and is involved in coping with chronic stress eventually leading to structural changes and prefrontal dysfunction [5–11]. Interestingly, the PFC of rodent models such as mice is limited in size, containing medial, orbitofrontal and cingulate areas, but probably lacking the equivalent of the primate dorsolateral PFC. In humans, the PFC can be considered to have evolved disproportionally large and it is thought to be the last region of the brain to gain full maturity [12, 13].

## Evolutionary view on PFC development

During mammalian evolution, the cerebral cortex not only increased in neuronal numbers and surface area but also acquired new cell types and cytoarchitectonic areas. Species-specific adaptations of prefrontal areas, steered by the environmental demands, can explain the differences in size of frontal areas over time. Among the most recent additions are several association areas, particularly the PFC, which has expanded enormously in primates culminating in humans [14]. In humans, the PFC occupies as much as about 30% of its surface. Although still debated, the human frontal lobe seems to have evolved three times larger than that of our closest living relatives, the great apes. In fact, it has been argued that the human brain possesses prefrontal regions that are both qualitatively and functionally exclusive [15]. It is, nevertheless, remarkable that we use the rodent model for most of the cellular and molecular neuroscientific studies, despite its lissencephalic brain which is clearly much simpler in both cytoarchitecture as well as function. A valid question still remains: *Do rodents have a prefrontal cortex?* [16, 17]. *And if we were to focus more on the evolutionary aspects of prefrontal development in terms of structural organization and function, should we not include longitudinal neurodevelopmental studies on more species* [18, 19]? Although the basic principles of cortical development may be similar in all mammals, the modifications of developmental events during millennia of primate evolution produce not only quantitative but also qualitative changes of its cellular structure and synaptic circuitry [13, 20]. The origin of species-specific distinctions can be traced either to the new or phylogenetically conserved genes that act at the time of the neural stem cell’s exit from the mitotic cycle and generate a different outcome, depending on the evolutionary context by interacting with a postmitotic neuron. Thus, the PFC as well as the Broca and Wernicke association areas in humans, which are formed in the frontal and temporal lobes, display a temporarily enriched gene expression pattern that is distinct from the mice or macaque cerebrum at the comparable prenatal stages (e.g., [21, 22]). More on evolution of the prefrontal cortex can be found in this volume, part I, chapter 1.

## The early stages of PFC development

### Genetic determination of the PFC

Still inside the womb, the generation of neural tissue (human, third gestational week) begins with the induction of ectoderm into neuroectoderm after which the neural tube will form through a process called neurulation [23]. The detailed analysis of a series of embryonic and fetal human postmortem brain tissue, as well as the evidence from experiments on animal models that range from rodents to nonhuman primates, showed that specific genes and regulatory elements are involved in evolutionary elaboration of the cranial part of the neural tube. More specifically, it is well documented that differential gene expression and the gradients of signaling molecules across the embryonic brain generate prospective subdivisions of the neocortex [24–29]. Work of Cholfin and Rubenstein in mice provide experimental evidence that the PFC can expand differentially and independently of the growth rate of the other areas [30, 31] and that its size can be regulated at early stages by the change of expression of specific growth factors before they receive the afferent axonal input [32]. Through regional specification in which the Fgf family plays a significant role, the (pre)frontal cortical area starts to expand [32]. The formation of the cytoarchitectonic map during evolution and individual development can be explained by the Protomap Hypothesis (PMH) of cortical parcellation [33]. This hypothesis postulates that intersecting gradients of molecules are expressed across the embryonic cerebral wall that guide and attract specific afferent systems to the appropriate position in the cortex where they can interact with a responsive set of cells [34]. The prefix “proto” indicates the malleable character of this primordial map, as opposed to the concept of equipotential cortical plate consisting of the undifferentiated cells that is eventually shaped and subdivided entirely by the instructions from those afferents [35, 36]. The PMH is at present universally accepted even by its initial opponents (e.g., [29]).

### Prefrontal expansion and lamination

The structural development of the various subdomains of the PFC is a meticulous process starting with a massive expansion of the most proximal part of the developing neural tube. The first step in the expansion of the cortical surface during development starts with an increase in the number of symmetrical divisions of neural stem cells in the ventricular zone (VZ) before the onset of neurogenesis and the formation of the subventricular (SVZ), intermediate (IZ) and subplate (SPZ) zones and cortical plate (CP) below the marginal zone (MZ) [33, 37–39], for review see [34]. This initial cortical expansion is also supported by experimental studies in mice [40–43] and provides an explanation for the massive increase in cortical surface area during both individual development and evolution.

**Fig. 1:**
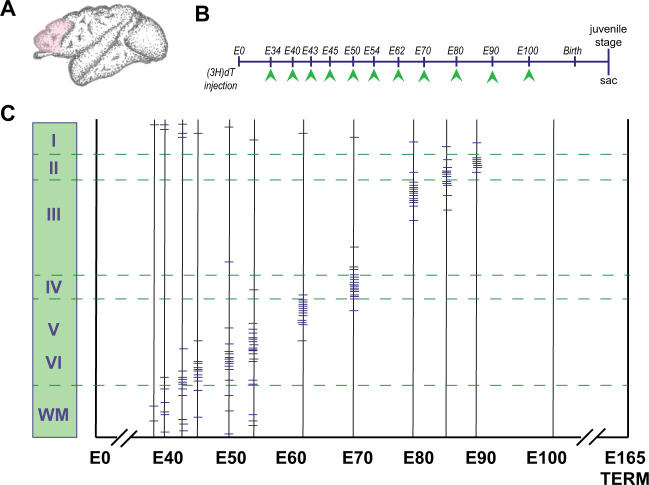
Prefrontal birthdating experiment in nonhuman primate. **A** Pen drawing of a macaque brain (side view) with the PFC indicated in pink. **B** Schematic overview of the time line for which [^3^H] thymidine ([^3^H]dT) injections were given at particular embryonic (E) time points indicated by green arrowheads. sac, sacrifice. **C** Relationship of time of origin and the final position of neurons destined for the PFC in macaque monkeys based on autoradiographic labeling of DNA replication by [^3^H] thymidine at various days of gestation (for details of the approach and methodology see Rakic [48]. Embryonic days are represented on the horizontal axis, vertical lines indicate the embryonic day on which an animal received a pulse of [^3^H]dT and the horizontal markers on the vertical lines represent the positions of heavily labeled neurons in the PFC. A schematic representation of the approximate position of layers I–VI and the white matter (WM) is indicated on the left (green rectangle). The data show that all neurons in PFC are generated between embryonic (E) day 40 and E90 within the 165-day-long gestational period in this primate species.

By the time the apical radial glial progenitors within the prefrontal subdomains start dividing asymmetrically, the number of neurons will increase rapidly and peaks between week 13 and 16 of gestation in human (E10-E15 rodents/E43-E50 primates), specifically in the dorsal telencephalon [44–47]. The labeling of dividing cells by the DNA replication markers tritiated thymidine (TdR) and bromodeoxyuridine (BrdU) showed that in nonhuman primate rhesus macaque, most cortical neurons, including those destined for the PFC, originate in the proliferative VZ near the cavity of the cerebral ventricle, between the 40th embryonic day (E40) and E100, during the 165-day-long gestational period in this species [48]. Genesis of neurons destined for the PFC is completed by E90, before completion of neurogenesis in the primary visual cortex at E90 (Fig. 1 and [49]). Through close interplay between cell-autonomous events and local as well as external cues, neurons generated close to the ventricle start migrating in radial columns [45, 46, 50, 51]. Gray matter continues to increase well into adolescence [52]. Astrocytes being the most abundant type of glial cells within cortical areas, are generated from the radial glial cells in the VZ and from the intermediate progenitors in the SVZ after the peak of neurogenesis [53]. The oligodendrocyte precursor cells, or OPCs, are generated within the medial ganglionic eminence and the anterior entopeduncular area and migrate toward the frontal cortical regions [54]. In the final stage of OPC production, this generation occurs in the cortical regions themselves. Microglia cells, on the other hand, are of mesodermal origin and migrate throughout the brain [55].

After the last cell division, postmitotic neurons migrate an increasingly long distance across the embryonic and fetal cerebral wall to their final positions in the cortex that develops below the pial surface [33, 56]. Although similar DNA labeling is not possible to perform in humans, examination of histological and Golgi silver impregnation methods of the embryonic and fetal human cerebrum indicate the existence of similar timing and sequence of these developmental events [37, 57, 58]. The pyramidal excitatory neurons born in the VZ and SVZ of the prefrontal subdomains, similar to other cortical areas, start to migrate radially toward the proper position in the CP under the influence of Fgfs [50, 59, 60]. Migrating neurons are guided over an increasingly long and curvilinear pathway by the elongated radial glial cell fibers that span the entire developing cerebral wall [61–63]. The radial glial processes that extend to the pial surface serve as a scaffold for the migrating neurons, which will settle themselves in an inside-out manner with the earlier-born neurons in the deeper layers and later born neurons in the more superficial ones [56, 64, 65]. Born in the ganglionic eminences, GABAergic interneurons migrate tangentially to the proper place within the prefrontal subdomains [66, 67]. Some recent findings in human and primates, such as the role of outer radial glia cells (oRGCs) and truncated glial cells, the diversity and complexity of cortical progenitors, the role of the subplate and the high specificity in axonal guidance events, again underline the complexity and evolution of cortical areas [68–75]. We now know from recent studies that it is the birth and migration of neurons derived from oRGCs that play a role in the development of the primary sulci (superior frontal, inferior frontal and precentral) in week 25–26 of gestation [23, 76]. After the process of migration is completed, RGCs retract their apical process and generate astrocytes and oligodendrocytes. In nonhuman primates and human, glial cells seem to somewhat outnumber neurons in the PFC, albeit with regional variation which is likely to contribute to the formation of secondary and tertiary gyri [77–81].

**Fig. 2:**
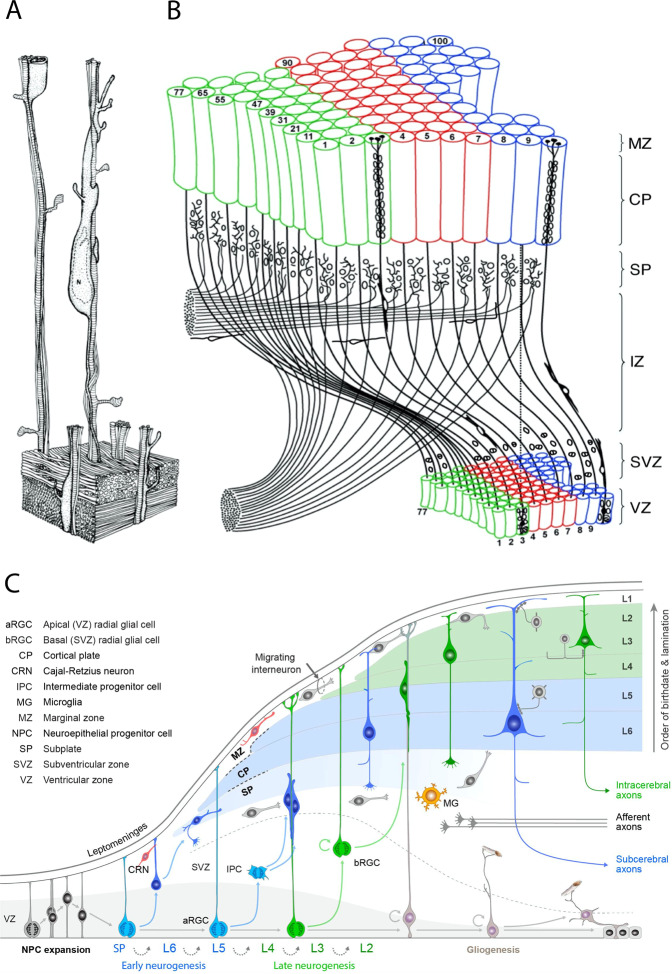
The evolution of corticogenesis. **A** Three-dimensional reconstruction of postmitotic neurons migrating along radial glial fibers, based on electron micrographs of semi-serial sections of the monkey fetal cerebral cortex with permission from Rakic [56]. **B** Representation of the radial unit hypothesis based on Rakic [33] with permission from Silver et al. [396]. **C** Illustration of the dynamics of major developmental events and diversity of progenitors involved in the development of primate cerebral neocortex based on studies of Rakic, with permission from Silver et al. [396].

Contrary to some initial concepts and theories [35, 36], embryonic VZ and CP are not uniform and equipotential. The enlargement and introduction of the new cytoarchitectonic areas has been explained by the Radial Unit Hypothesis (RUH). According to this hypothesis, increasing the size and proliferative capacity of the neuronal stem cells in the proliferative zone  enables initial enlargement of the cortex as well as formation of the distinct anatomical and functional cytoarchitecture areas in the mammalian evolution [33]. According to the RUH, tangential (horizontal) positions of cortical neurons are determined by the positions of their precursor cells, now called stem cells in the VZ, while their radial (vertical) position in the overlying cortex is determined by the time of their origin (Fig. 2). Therefore, the addition of the number of the radial columns increases the size of the cortical surface, whereas the number of cells within the columns determines its thickness.

### Differentiation and synaptogenesis

**Fig. 3:**
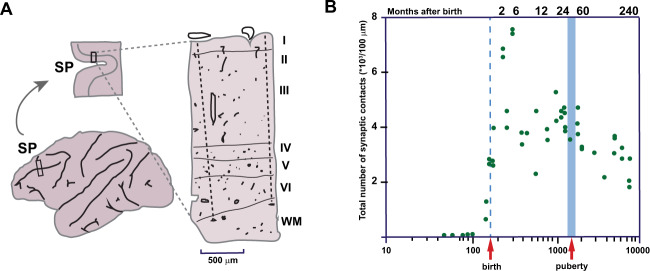
Primate synaptogenesis in the PFC assessed by quantitative electron microscopy. **A** Schematic representation of the site of the block dissection from the depth of the Sulcus Principalis (SP). On the right: The section of the cortex in the SP showing the vertical (radial) probes across layers I–VI, which were examined by electron microscopy. **B** The total number of synaptic contacts in each vertical probe as represented by the green dots. The semi-log plot in abscissa represents the number of days after conception. Adapted from Bourgeois et al. [49].

**Fig. 4:**
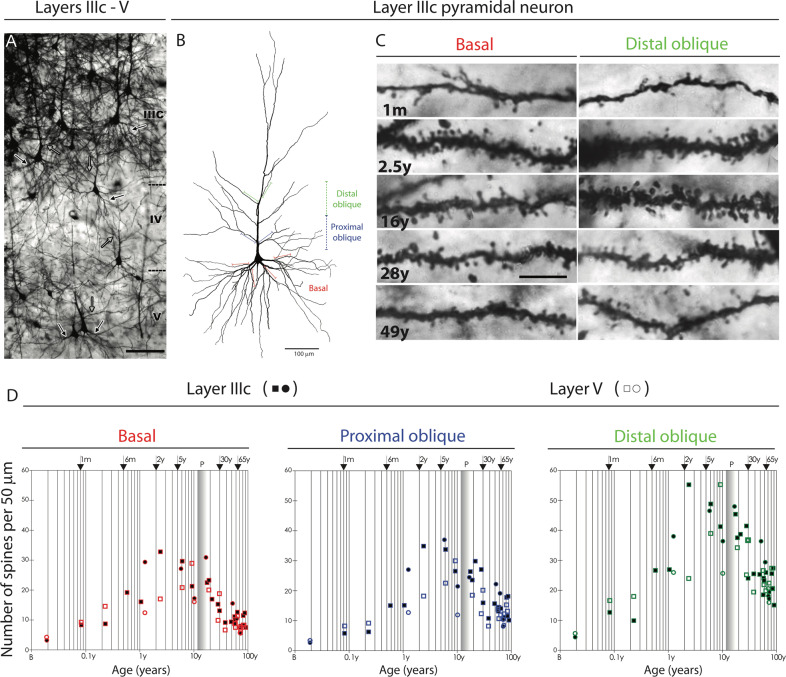
Development of dendritic spines on layer IIIC and layer V pyramidal neurons in the human PFC. **A** Low-magnification photograph of the rapid Golgi-impregnated layer IIIc and V pyramidal cells in the dorsolateral PFC of a 16-year-old subject. **B** Neurolucida reconstruction of layer IIIc pyramidal neuron of a 49-year-old subject showing distal oblique (green), proximal oblique (blue) and basal dendrites (red). **C** Representative high-power magnification images of rapid Golgi-impregnated layer IIIc pyramidal neurons in a 1 month old infant, 2.5-year-old child, and 16-, 28-, and 49-year-old subjects. **D** Graphs representing number of dendritic spines per 50-μm dendrite segment on basal dendrites after the first bifurcation (red); apical proximal oblique dendrites originating within 100 μm from the apical main shaft (blue); and apical distal oblique dendrites originating within the second 100-μm segment from the apical main shaft (green) of layer IIIc (filled symbols) and layer V (open symbols) pyramidal cells in the human dorsolateral PFC. Squares represent males; circles represent females. The age in postnatal years is shown on a logarithmic scale. From Petanjek et al. [12].

After neurons assume their final position, they begin to differentiate further and form synaptic connections. In humans, between 17 and 50 weeks of gestation (first to fourth postnatal week in rodents), the pyramidal and interneurons in the various cortical layers of the PFC will further mature and differentiate [82, 83]. The basal and apical dendritic length will increase, the spines will further develop, specifically in layer III and V, and their axons will extend to other cortical and subcortical targets [82, 84]. This is also the case for the inhibitory network where the interneurons mature extensively with a sharp increase in the dendritic spine formation but also in terms of their intrinsic as well as their network properties as was shown in mice [85, 86]. Prefrontal synaptogenesis starts prenatally and peaks postnatally followed by a process called pruning or refinement of synaptic connections, the removal of unused synaptic contacts [87]. When neurites to and from the PFC reach their final target position, an immature synapse is generated under the influence of, among others, cell adhesion molecules and reelin [88, 89]. Epigenetic regulatory factors such as microRNAs (miRNAs) play an important role in this process by modulating dendritic and synaptic maturation [90, 91]. The tempo and kinetics of synapse formation in the primate PFC closely resemble those described for other areas [92]. In young primate embryos, a precortical phase (E47-E78) is described when synapses are found only above and below, but not within, the CP. Following that, there is an early cortical phase, from E78 to E104, during which synapses accumulate within the cortical plate, initially exclusively on dendritic shafts. The next rapid phase of synaptogenesis begins at 2 months before birth and ends approximately at 2 months after birth, culminating with a mean density of 750 million synapses per cubic micrometer. This accumulation is largely accounted for by a selective increase in axospine synapses in the supragranular layers. Therefore, the early childhood PFC contains a 2–3 fold higher density of dendritic spines compared to the adult PFC. The period of overproduction of synapses is followed by a protracted plateau stage that lasts from 2 months to 3 years of age when synaptic density remains relatively constant. In humans, the PFC synaptic density spikes around 3.5 years of age (~4th postnatal week in rodents), which is relatively late compared to other cortical areas and almost double the net density of the adult PFC [82, 93, 94]. Examination of the course of synaptogenesis in the macaque PFC, by detailed quantitative electron microscopic analysis, showed that the number of synaptic contacts is initially grossly overproduced before declining to the normal adult level (Fig. 3 and [49]. Likewise, the axons of the corpus callosum, as well as other large axonal tracts in the macaque cortex, including PFC, are grossly overproduced before decreasing to the adult level [95–97]. A subpopulation of GABAergic neurons in the subplate zone also form transient synapses that are eventually eliminated [98, 99]. The period of synaptic decline in human PFC, which starts during childhood, is initially dramatic and continues during adolescence and extends at a slower, but statistically significant rate into the 3rd decade of life (Fig. 4 and [12]).

The finding that synaptic density in the cerebral cortex is relatively stable from early adolescence through puberty (the plateau period) is indicative that in primates the final synaptic pattern is the result of selection and refinement of their higher number during the formative years when learning experiences are most intense. These discoveries led to the proposal that the Selective Elimination Hypothesis is a mechanism for tuning synaptic connections by interaction with the environment during the period of most intense learning [92]. These days, selective elimination or stabilization is commonly called “pruning”, and this refinement of the differentiating cortical network via pruning of dendritic branches, and/or efferent/afferent projections, is an important process to fine-tune the meticulous intricate prefrontal network [100, 101]. Within the rodent and primate PFC, this process of synaptic pruning, which is most dramatic in layer III, continues well into adolescence leading to a long-lasting decline in synaptic density across PFC subdomains [82, 102, 103]. It was furthermore discovered that major neurotransmitter receptors are also initially overproduced in all eight primate prefrontal regions examined [104, 105]. Moreover, during childhood the PFC myelination process starts (white matter volume increase) which continues into adulthood [106, 107].

### Getting connected

The prolonged maturation of the PFC depends largely on the coordinated action of various external factors. Most neurotransmitter projections arrive in the prefrontal subdomains in two streams: within the marginal zone (MZ) and within the subplate zone (SPZ) which is thicker in the PFC compared to other cortical areas. A major change in development, which likely signals roots in the evolution of the cortex, is in the specificity in neurotransmitter systems alongside a boost in receptor type heterogeneity in primates and human [108–112]. In humans the thickening of the PFC subplate has evolved tremendously, suggesting playing a role in the extensive prefrontal circuitry [71, 102]. Vice versa, the multitude of pyramidal neurons in the various layers and PFC subdomains will connect to other cortical and subcortical targets by extending their axons, once they have reached their final position in the PFC (human: birth till end of first year/rodent first 2 postnatal weeks). The intricate timely integration of all these neurotransmitter systems is essential for prefrontal functioning. In this way, a unique and higher-order functional network capable of emotional processing and complex cognitive abilities is established.

#### Developing PFC connections - from the neurotransmitter perspective

##### Serotonin

The brain matures from the brainstem to the more frontal cortical regions, and it is therefore not surprising that serotonergic projections from both the dorsal as well as the medial Raphe nuclei (DRN, MRN respectively) are among the first to emerge and are set towards cortical regions where they arrive in the PFC around E16/E17 in rodent and week postnatal 10–13 in humans [113–116]. Most of the work on molecular and cellular underpinnings of serotonin functioning and guidance during early development have been investigated in rodents, although it is clear that the specificity of serotonergic prefrontal connectivity in primates and human increase tremendously in regional specificity [109]. In mice, the serotonergic projections toward the forebrain are predominantly guided by the Epha5/ephrina5 interaction of guidance cues [117]. Of note, early in the development of serotonergic signaling, molecules such as receptors and transporters are already expressed in the forebrain and an exogenous placental source of 5-HT has been considered to direct cortical development even before raphe-derived projections have reached the forebrain [118–121]. Once the serotonergic projections have arrived within cortical areas, they are able to make contacts with Cajal Retzius cells within the MZ, thereby raising the possibility of playing a role in neuronal migration [122–124]. It has become widely accepted that serotonin exerts a significant trophic and modulatory function in neurodevelopmental processes such as proliferation, migration and differentiation in cortical areas, including the PFC [119, 123, 125–129].

##### Noradrenalin

The Locus Coeruleus (LC) in the brainstem sends out its noradrenergic axonal projections to the PFC as early as E16/17 (rodent) and week 10–13 in human [130–132]. It appears to be a heterogeneous set of neurons innervating all aspects of the PFC subdomains [133–135]. Noradrenergic projections arrive in cortical areas before all cortical neurons have finished migrating and have adopted their final appearance [136]. During the embryological development of prefrontal areas specifically, noradrenaline plays a role in cell division, neuronal migration, differentiation as well as synaptogenesis [137–141]. Like serotonin, noradrenergic axons make contact with the Cajal Retzius cells in the marginal zone, suggesting a role in the laminar formation of cortical regions [132, 142, 143]. In addition, noradrenalin seems to have an effect on the development of dopaminergic projections in the PFC by providing a dopamine reuptake mechanism through the noradrenalin transporter [144, 145] as well as on GABAergic signaling in the PFC [146, 147]. Recent studies of rat and primate PFC showed that the α2-adrenoceptor and muscarinic M1 receptor modulate working memory via KCNQ potassium channel [148–151]. Reciprocal direct connections from the mPFC to the LC mature over time, and this system is involved in a variety of behaviors such as memory formation, attention, arousal, vigilance and coping with stress [152–154].

##### Dopamine

A subset of the medial part of the ventral tegmental area (VTA) starts to project to prefrontal subdomains around E15/E16 (rodent) and week 10–13 in human [155–158]. Steering dopaminergic projections from the VTA via the medial forebrain bundle toward forebrain regions mostly depend on a coordinated action of the guidance molecules Dcc and Netrin-1 mediated by microRNA miR-218 control of Dcc expression in the VTA [159–161], while Semaphorin3F is orchestrating their fasciculation, rostral growth and targeting within the various mPFC subdomains [156]. The dopaminergic innervation of the mPFC in rodent surges during adolescence hallmarked by massive changes in the organization, shape and density of the dopaminergic fibers [162–164]. A similar surge in regional-specific dopaminergic connectivity to the PFC can be observed in primates, including human modulating local microcircuits [165–169]. Of note here is that some of these dopaminergic neurons projecting to the various PFC subdomains are capable of co-releasing glutamate as well and have an exclusive excitatory effect on the GABAergic interneurons in the various layers of the PFC [170–173]. Eventually, the mature mesoprefrontal system is involved in attention, behavioral flexibility, action planning, sustainability of motivational and affective states, working memory and memory consolidation which is mediated in parallel by catecholaminergic pathways [169, 174–178]. In many neurodevelopmental disorders (NDDs) the developing dopamine system is affected playing a role in the diverse symptoms of these disorders [179].

##### GABA

Most of the GABAergic interneurons are born in the ganglionic eminences of the ventral telencephalon and migrate tangentially to the proper cortical areas and layers to form a network with the radially migrated pyramidal neurons [180–182]. Initially being excitatory through the GABA_A_ receptors expressed on radial glia cells and migrating interneurons, GABA plays a role in proliferation, migration and synaptogenesis [183–186]. It has furthermore been shown that dopamine and GABA interactions can influence these processes [187, 188]. Around the second postnatal week in rodents (~first postnatal week in human), the depolarizing effect slowly transitions into an inhibitory net effect depending on place and time [189, 190]. A remarkable feature in GABA signaling from an evolutionary perspective is that in nonhuman primates and human there seems to be a cell-type specific expression of the GABA transporter GAT-1 in early childhood [191, 192]. Furthermore, it appears that nonhuman primates and humans have distinct populations of GABAergic neurons which originate in proliferative zones of the dorsal telencephalon [193, 194].

##### Glutamate

There are various sources of glutamatergic input projections including a subset of (non-)dopaminergic VTA neurons to GABAergic interneurons in the PFC [170–173]. The most prominent monosynaptic inputs of the PFC are derived from hippocampus, mediodorsal (MD) thalamus and amygdala [195–201]. In fact, the medial pulvinar part of the medial thalamus or PM, which evolutionary expanded alongside the association cortex in nonhuman primates and human, is characterized by a distinct prefrontal glutamatergic connectivity that seems to play a significant role in NDDs [202]. A multitude of cortical and subcortical targets are progressively innervated by developing glutamatergic projections from the PFC itself such as the various thalamic regions. Recently it was found that retinoic acid (RA) plays a critical role in PFC development and specifically in this thalamus-prefrontal connectivity [203]. The PFC furthermore sends out glutamatergic afferents to the VTA as well as to the nucleus accumbens modulating dopaminergic signaling [204–206]. In addition, DRN serotonergic neurons are controlled by glutamatergic projections from the PFC [152, 207–209].

##### Acetylcholine

Around birth, the numerous cholinergic projections arising from the basal forebrain nuclei innervate the primary cortical regions where they influence cortical ultrastructure [210–213]. Acetylcholine modulates primarily the prelimbic subdomain of the PFC during development targeting GABAergic interneurons [210]. But even before the cholinergic projections arrive in the cortical areas, the nicotinic and muscarinic receptors are expressed on neural progenitors playing a role in proliferation/differentiation and axonal guidance events [214–217]. In the PFC of nonhuman primates, the muscarinic M1 receptors modulate working memory via KCNQ potassium channels [151]. Alongside, a transient expression of the enzyme acetylcholinesterase seems to play a role in the thalamocortical circuit formation [218–220]. Within the PFC, the cholinergic innervation initially terminates in layers III and IV slowly losing laminar preference over time [221–223]. Key developing PFC circuitry is shaped by acetylcholine, and PFC pyramidal neurons depend on its proper signaling in terms of dendritic branching, spine formation and synaptogenesis [224–226].

#### Convergence of developing transmitter systems within the PFC

There is, furthermore, ample evidence now that during embryonic development there is convergence of the various neurotransmitter signaling pathways and influence each other’s development and functioning [153, 227–231]. These neurotransmitter systems can act as neurotrophic factors steering various neurodevelopmental events in their target areas. Serotonergic and dopaminergic markers are jointly present in their developmental origins, guidepost areas, as well as within the subdomains of the PFC, which is important for their intricate interaction later in life to establish higher cognitive functions [209, 232–234]. The same holds true for noradrenergic and dopaminergic projections towards forebrain regions as well as dopaminergic-glutamatergic and dopaminergic-cholinergic interactions controlling PFC maturation and functioning [227, 228, 231]. These neurotransmitter projections initially innervate prefrontal regions via two parallel paths; one via the subplate and one via the marginal zone where the Cajal Retzius cells reside [235]. Being in close proximity of the CR cells, it is likely that volume transmission is used to release the neurotransmitter. Receptors, transporters as well as synthesizing enzymes are already expressed (~E10 rodent and week 4–5 human) even when the actual axonal projections have not yet arrived in the PFC [236–240]. In fact, neurotransmitter receptors are found to be expressed by progenitor cells throughout development [231]. External neurotransmitter sources, such as the placenta, can play a role in this early shaping of cortical areas [114, 118, 241, 242]. All this is especially important in light of (anti-depressant) use of pharmacological drugs during pregnancy as they can interfere with these early signaling pathways and hamper the structural development of brain areas including the PFC.

## PFC cognitive development

The PFC, as the seat of our higher-order cognitive functions, continues to develop into adulthood [52, 243]. It is among the latest brain regions to fully mature in humans as well as rodents [106, 159, 244, 245]. The primary somatosensory cortex, as well as the primary motor cortex, mature earlier, however the dendritic trees and the density of spines within the subdomains of the PFC seem to be more complex [246–249]. Cognitive abilities are shaped by experience over time and seem to be in synchrony with PFC structural changes such as synaptogenesis and pruning [250]. Following the ‘use it or lose it’ principle, the developing PFC dynamically rearranges incoming and outgoing wiring depending on usage and need [12]. Specific for the PFC, the non-coding microRNAs mir-128b and mir-30a-5p have shown to be involved in prefrontal-dependent cognitive maturation by affecting epigenetic mechanisms [251, 252]. The constantly developing cognitive and executive capabilities occur parallel to the neurophysiological changes within the PFC and its connected areas and seem to reach a plateau in teenagers (around 12 years in human, around P50 in rodents) [253]. Adolescence is typically characterized by changes in social interactions and cognitive abilities in order to gain independence and adult skills and competences [245]. In nonhuman primates this is characterized by risk-taking, novelty seeking, and increased vigilance; whereas in rodents by play behavior, increased exploratory activity and impulsivity are peaking [245]. Higher order cognitive functions, in which PFC plays a prominent role, such as language and intelligence, continue to develop into adulthood [254, 255]. More on the role of the PFC in cognitive control and executive functioning can be found in this volume in the reviews by Robbins and Friedman (I.6) and Menon and D’Esposito (I.7).

## PFC development and mental illness

**Fig. 5:**
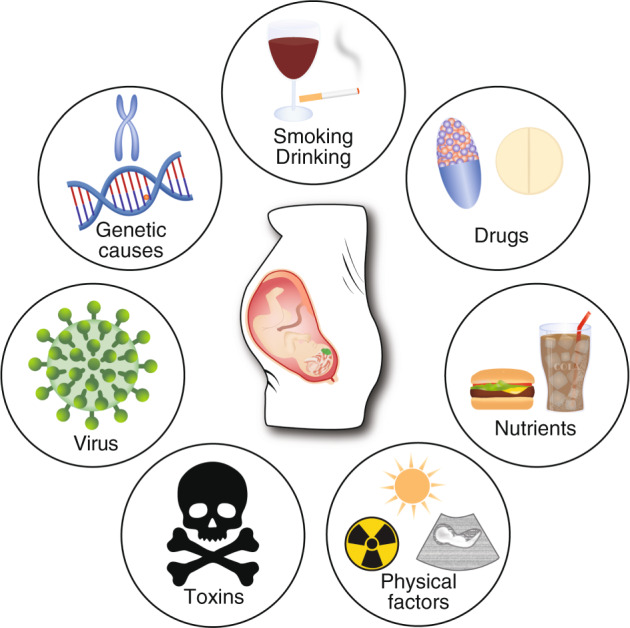
Risk factors in PFC development. Schematic overview of genetic and environmental risk factors during pregnancy to be the possible cause of NDDs (Clockwise: Genetic causes, Smoking, Drinking, prescription or recreational Drugs, certain combination of Nutrients, Physical factors such as UV, ultrasound or various radiations, Toxins, Virus infections). Possible causes for NDDs include specific genetic or environmental factors as well as a combination of both.

Although stress-induced structural changes in the PFC are equally important in their contribution to the pathophysiology of neuropsychiatric conditions [256–262] (see also part III of this volume), we focus in this paragraph on particular risk factors involved in the onset of NDDs) in which PFC functioning is affected. It has been speculated that, as the PFC takes so long to fully mature, it also has the largest critical window of all developing brain areas. The various risk factors, either genetic or environmental, can hamper the intricate developmental events and pose a risk in developing NDDs (Fig. 5).

### From a genetic point of view

The group of patients having a NDD is enormously heterogeneous. The genetic causes underlying NDDs are diverse ranging from single gene mutations, copy number variations to whole‐chromosome aberrations [263]. Even with monogenic causes, the severity and comorbidity of the symptoms can vary tremendously and neurological/neuropsychiatric symptoms are often accompanied by additional clinical features such as maldevelopment of organ systems. But there are also some clear examples of environmental risk factors that specifically hamper PFC development resulting in behavioral and cognitive deficits. Below we list specifically those NDDs where the structural development of the PFC is clearly affected.

#### Monogenic causes

Many of the Mendelian monogenic NDDs are characterized by intellectual disability and behavioral problems due to, in part, an altered prefrontal functionality. Fragile X syndrome (FX) is a NDD where the causative gene, Fragile X Mental Retardation Protein (FMRP), is completely absent causing a plethora of developmental abnormalities [264, 265]. It is clear that in FX the many behavioral and cognitive deficits can be attributed, at least in part, to prefrontal dysfunction. Some of these aspects could be rescued in an animal model where FMRP production was initiated in the mutant PFC [266]. In Rett syndrome, a severe NDD with specific cognitive and behavioral features, there is a strong PFC hypofunction with structural abnormalities [267–269]. Restoring Mecp2 levels within the PFC in mice via state-of-the-art techniques such as CRISPR-Cas9 or DREADDS can restore some of the endophenotypes such as social recognition deficits or long-term retrieval of auditory conditioned fear [267, 270, 271]. Other monogenic syndromes like Kleefstra, KBG, WitKoS, Angelman, Coffin-Sirris, Rubinstein-Taybi, Phelan-McDermid, Smith–Magenis Syndrome and Kabuki syndrome also have a clear prefrontal component in their behavioral and cognitive phenotype [272–280]. For some of these syndromes it has recently been shown that deficits in the structural development of the PFC underlie these problems [281–287].

#### Chromosomal abnormalities

In all human chromosomal aberration syndromes, including trisomies, monosomies (e.g., Turner syndrome, monosomy 1p36), polyploidies, disomies and imprinting errors or sex chromosome anomalies, structural abnormalities of (pre)frontal as well as many other areas are common [61, 288–292]. Trisomy of chromosome 21 or Down syndrome can be considered a NDD with significant developmental deficits. Cognitive abilities are affected due to a developmental delay including the maturation of brain areas such as the PFC [293]. Particular neurodevelopmental events are delayed in forebrain regions such as neurogenesis, migration and synaptogenesis eventually resulting in altered prefrontal circuitry [294–296]. Williams (WBS or WS) syndrome is a rare NDD with a deletion of approximately 25 genes on chromosome 7 and characterized by an unusual sociability and cognitive deficits [297]. The structural organization of prefrontal pyramidals, specifically their density and dendritic arboring, is severely affected [298, 299]. Prader-Willi syndrome (PWS) is a disorder in which imprinted genes on chromosome 15 are affected and is characterized by increased volume of prefrontal subdomains important in the reward circuitry [300, 301]. In the 22q11.2 deletion syndrome (or DiGeorge/Velo-Cardio-Facial syndrome), individuals are characterized by loss of executive function and working memory alongside other cognitive problems and MRI studies showed a clear loss of volume of the various PFC subdomains [302–307]

### From an environmental point of view

#### Food/drugs

One of the most studied risks during pregnancy is the composition of our diet. Many food-derived molecules can reach the unborn baby in one form or another, and therefore could directly or indirectly influence brain development when crossing the immature blood-brain barrier and potentially affect PFC development [308]. It is therefore important to realize that with the change of our diet through the ages, having become more processed and high-fat and high-sugar in contents, this can have a dramatic effect on the development and functioning of the PFC. Particular consumption of high-fat and/or high-sugar during pregnancy, childhood and adolescence can result in structural changes in the PFC and deficits in executive functioning [308–311]. Maternal metabolic disorders including diabetes and obesity can pose another threat to the unborn child as placental dysfunctioning alters the prenatal exposure to nutrients and toxins [312–314]. In the early ‘70s it was found that women who abused alcohol during pregnancy may deliver children with severe developmental delays, smaller brains and cognitive problems called Fetal Alcohol Syndrome (FAS) [315]. These children often have various conditions that are collectively known as Fetal Alcohol Spectrum Disorder (FASD), which includes FAS and a condition known as Alcohol-related Neurodevelopmental Disorder (ARND). There is a clear correlation of children with FAS and prefrontal executive functioning [316]. MRI studies showed reductions in brainstem as well as cerebellum volume in a primate FAS model and a sex-dependent change in functional connectivity and metabolism in prefrontal areas in a rat FAS model [317, 318]. Structurally, the prefrontal cortical thickness is affected after prenatal alcohol exposure and it matures with a smaller number of excitatory neurons and more GABAergic ones disrupting the excitatory/inhibitory balance severely [319–321]. Similar structural and behavioral defects of the PFC can be observed in kids with prenatal exposure to opioids, cocaine, amphetamines and other drugs-of-abuse [322–330]. Similarly, we can find lead and other pollutants to be damaging to the developing brain and PFC [331–334]. Another field of recent study is the perinatal exposure to pharmaceuticals given to treat the pregnant mother. Perinatal HIV infections can alter the course of brain development (see below), on the other hand perinatal exposure to antiretroviral drugs such as Efavirenz (EFV) to treat HIV leads to an altered prefrontal cytoarchitecture [335, 336]. Although maternal stress itself can be detrimental to brain development in general and the developing PFC in particular (for review see [125]), treatments against maternal depression such as SSRIs can cause substantial structural damage to prefrontal subdomains [125, 128].

#### Viral infections

Traditionally, pregnant women were warned for TORCH (TOxoplasmosis, Rubella, Cytomegalovirus, and Herpes simplex viruses type 1 and 2) infections especially during the first two trimesters of pregnancy as they were shown to cause severe congenital abnormalities [337]. Later, the O was referring to Other infections such as syphilis, varicella-zoster, and parvovirus. Zika viral infection during pregnancy can cause microcephaly including severe structural damage to the prefrontal areas [338–340]. The Zika virus is able to infect neuroepithelial stem cells and cortical radial glial cells and to a lesser extent postmigratory neurons causing structural disorganization in these cells eventually leading to cell death [341–343]. Even a postnatal viral infection can lead to postnatal meningitis and neurodevelopmental problems due to structural and functional damage of frontal areas [344]. In the recent SARS-CoV-2 or COVID-19 viral outbreak, similar structural damage of frontal cortical areas could be observed, most likely due to an inflammatory response in which parenchymal cells and the choroid plexus are involved [345–347]. Little is known however on the short- and long-term effects of a COVID-19 infection during pregnancy and the possible neurodevelopmental changes it can make during corticogenesis leading to NDDs [348].

#### Other perinatal causes

Multiple fetuses per pregnancy, intrauterine growth restriction (IUGR, due to placental failure other than by causes described above), X-ray, UV, nuclear or cosmic radiation, (ultra)sound as well as high temperature, preterm birth or hypoxia whether or not by traumatic causes can pose serious threats to proper corticogenesis as well [349–356]. Recently it has become clear that IUGR is associated with an increase in impulsive behavior due to an altered dopamine signaling in the PFC [357, 358]. Perinatal hypoxia can furthermore change the expression of cytokine and ceramide metabolism genes in the PFC and hampers cognitive functioning in later life [359, 360]. In preterm birth, changes in white and gray matter including reductions in cortical surface area and cortical thickness of the PFC are described [361–364]. Disruptions in PFC network activity often further aggravates the already compromised neurocognitive development in these children [365, 366].

### The multifactorial view

It is now generally accepted that the etiology of many NDDs is considered to be multifactorial. Often, comorbidity of two or more NDDs is observed. Variable environmental exposure to risk factors combined with variable genetic background makes it hard to pinpoint possible causes. Yet, as the PFC takes the longest to fully mature, we can argue that it is most vulnerable to any risk factor when presented early enough. We will here review three of the ‘classical’ NDDs that are considered to be multifactorial in their onset with affected PFC development. Intellectual disability (ID) is an umbrella term and is a comorbidity of many of the described NDDs and will therefore not be discussed separately.

Autism spectrum disorder (ASD) is an example heterogeneous NDD characterized by impaired communication and social interaction accompanied with repetitive behavior and stereotyped interests. It has been described that the PFC of individuals with ASD show structural and functional changes, specifically in the ACC, oFC and lPFC [367, 368]. The number of neurons (but also their size), specifically the chandelier cells, basket cells and other parvalbumin-expressing interneurons, is decreased in the PFC [369–371]. There are also indications that serotonergic signaling is affected during PFC development [372]. In the first couple of years of life there is a prefrontal hyperconnectivity in children with ASD followed by a hypoconnectivity resulting in a ‘disconnection’ with other cortical areas involved in higher-order associative processing [373–376]. The short- and long-range prefrontal axons, particularly those from the ACC, are affected in their guidance to subcortical targets and may underlie the network disruption characteristic for ASD [377, 378]. A new and interesting finding is that there appears to be a major change in the levels of various metabolites in the PFC of autistic individuals [379].

Attention deficit/hyperactivity disorder (ADHD) is a NDD characterized by signs of inattention, impulsivity, and hyperactivity [380, 381]. Control processes mediated by the PFC are hampered. Imaging studies have shown the PFC to be thinner in ADHD individuals, thereby hampering proper maturation and prefrontal connectivity leading to attentional dysfunction [382, 383]. Loss of catecholaminergic innervation underlies the most important ADHD symptoms [381].

Epilepsy, specifically childhood frontal lobe epilepsy (FLE) has various clinical outcomes but most often resulting in multi-cognitive symptoms [384–387]. Elevated prefrontal oscillations and hippocampal-prefrontal theta coherence could be observed after FLE [388]. And eventually the FLE seizures can cause structural and functional damage of the prefrontal areas including altered short-term plasticity [389, 390]. Important is also that the anti-epileptic drugs, given at young age or during pregnancy, can have major neurodevelopmental implications as well. These drugs act upon the major neurotranmitter and second messenger systems including ion channels, thereby affecting neurodevelopmental events [391–393].

## Future research directions

The multidisciplinary nature of the field of developmental neurobiology has made enormous progress in recent years. The combination of classical (immuno)histological techniques with physiological, behavioral and high power molecular approaches such as large-scale genome-wide (single-cell) transcriptome and epigenome profiling studies have brought us an enormous amount of insight and resolution into the development and evolution of developing brain areas, specifically the PFC and their role in the onset of NDDs [21, 394]. Advances in molecular labeling and imaging techniques have added to this understanding [4, 395]. But maybe the most exciting field is the rapid emergence of stem cell approaches such as the generation of brain organoids that have led to some tremendous breakthroughs. A dazzling number of studies in gyrencephalic species have led to scientific breakthroughs and the description of novel types of cortical progenitors, including the basal and outer RGCs, both of which have been linked to cortical expansion and folding. Early features of corticogenesis can be recapitulated reliably; however the later stages in development still need to be optimized.

It has become clear that most neurotransmitter systems play neurotrophic roles during neurodevelopment as well. More holistic studies into the extrasynaptic neurotrophic functions of neurotransmitters during prefrontal development might also provide more understanding of their potential roles in the etiology of NDDs and eventually will enable us to design critical developmental windows in which we may be able to intervene. In the future, more longitudinal as well as interspecies studies will be needed to corroborate our understanding of prefrontal development.

Abnormal PFC development may lead to a variety of behavioral and cognitive problems inherent to psychiatric disorders including NDDs. In order to create tailored interventions targeted to the specific genetic syndromes, there is a strong need for research into the specific developmental and behavioral aspects accompanying these syndromes. A better understanding of the underlying neurodevelopmental and biological mechanisms will open doors to investigate the possibility of therapeutic (early/preventive) interventions and subsequent improvement of care.
